# Cingulin–nonmuscle myosin interaction plays a role in epithelial morphogenesis and cingulin nanoscale organization

**DOI:** 10.1242/jcs.262353

**Published:** 2024-09-25

**Authors:** Florian Rouaud, Marine Maupérin, Annick Mutero-Maeda, Sandra Citi

**Affiliations:** Department of Molecular and Cellular Biology, University of Geneva, 30, Quai E. Ansermet, 1205 Geneva, Switzerland

**Keywords:** Cingulin, Nonmuscle myosin, Epithelial cells, Hearing, Cyst morphogenesis

## Abstract

Cingulin (CGN) tethers nonmuscle myosin 2B (NM2B; heavy chain encoded by *MYH10*) to tight junctions (TJs) to modulate junctional and apical cortex mechanics. Here, we studied the role of the CGN–nonmuscle myosin 2 (NM2) interaction in epithelial morphogenesis and nanoscale organization of CGN by expressing wild-type and mutant CGN constructs in CGN-knockout Madin–Darby canine kidney (MDCK) epithelial cells. We show that the NM2-binding region of CGN is required to promote normal cyst morphogenesis of MDCK cells grown in three dimensions and to maintain the C-terminus of CGN in a distal position with respect to the ZO-2 (or TJP2)-containing TJ submembrane region, whereas the N-terminus of CGN is localized more proximal to the TJ membrane. We also show that the CGN mutant protein that causes deafness in human and mouse models is localized at TJs but does not bind to NM2B, resulting in decreased TJ membrane tortuosity. These results indicate that the interaction between CGN and NM2B regulates epithelial tissue morphogenesis and nanoscale organization of CGN and suggest that CGN regulates the auditory function of hair cells by organizing the actomyosin cytoskeleton to modulate the mechanics of the apical and junctional cortex.

## Teaser

**Highlighted Article:** The interaction between cingulin and nonmuscle myosin regulates cyst formation of MDCK cells and maintains the nanoscale organization of cingulin. The deafness-associated cingulin variant affects tight junction membrane mechanics.

## INTRODUCTION

The apical junctional complex (AJC) of epithelial cells, comprising tight junctions (TJs) and adherens junctions (AJs) (Farquhar and Palade, 1963), is critical in the function of epithelial tissues, as it establishes and maintains selective tissue barriers (TJs) and tissue integrity (AJs), and participates in signalling to regulate cell polarity and tissue morphogenesis (Macara et al., 2014; Flinois et al., 2024). Epithelial tissues mediate tissue and organ functions by responding to external and internal physiological and pathological stimuli. This is achieved through different mechanisms, including the sensing and transduction of mechanical signals, which occurs at the AJC and at the membrane cortex (Charras and Yap, 2018; Citi, 2019). The association of the AJC with the actomyosin cytoskeleton is essential not only for mechanosensing, but also for the organization and function of the AJC. For example, the peri-junctional actomyosin ring associated with AJs (Ebrahim et al., 2013; Heuze et al., 2019) is critically important both for the regulation of TJ barrier function and for AJ resistance to mechanical stress (Yap et al., 2017; Buckley and Turner, 2018; Citi, 2019; Horowitz et al., 2023; Citi et al., 2024). However, little is known about the mechanisms of linkage of actin and myosin isoforms to the cell cortex and to the AJC, and how these affect epithelial morphogenesis and mechanobiology and junction architecture.

Currently available data indicate that TJs are connected to the actomyosin cytoskeleton primarily through ZO-1 (also known as TJP1) and cingulin (CGN) (Fanning et al., 1998; Citi et al., 2012; Van Itallie and Anderson, 2014; Belardi et al., 2020; Rouaud et al., 2020, 2023; Citi et al., 2024). ZO-1 is an actin-binding mechanosensing protein, which can exist in stretched and folded conformations, depending on actomyosin-generated force and interaction with ZO-2 (TJP2) (Spadaro et al., 2017), as well as phosphorylation and multivalent interactions (Beutel et al., 2019). ZO-1 stretching affects its interaction with signaling proteins, such as the transcription factor DbpA (Spadaro et al., 2017), and is required for TJ assembly and embryo morphogenesis (Beutel et al., 2019; Schwayer et al., 2019). CGN is an adaptor TJ protein (Citi et al., 1988) that is recruited to TJs by ZO-1 (D'Atri et al., 2002; Umeda et al., 2004; Vasileva et al., 2022) and tethers nonmuscle myosin 2B (NM2B; heavy chain encoded by *MYH10*) to ZO-1 at TJs (Rouaud et al., 2023) through interactions mediated by the C-terminal parts of their respective coiled-coil rod domains. CGN can also interact *in vitro* with nonmuscle myosin 2A (NM2A; heavy chain encoded by *MYH9*), albeit with lower affinity (Cordenonsi et al., 1999; Rouaud et al., 2023). The ZO-1–CGN–NM2B complex connects TJs to the actomyosin cytoskeleton, and the integrity of this complex not only promotes the stretching of ZO-1 and its accumulation and stabilization at TJ (Vasileva et al., 2022; Rouaud et al., 2023), but also regulates the mechanics of the junctional and apical membranes, as it promotes increased TJ membrane tortuosity and apical membrane cortex stiffness in Madin–Darby canine kidney (MDCK) cells (Rouaud et al., 2023). However, it is not clear if and to what extent the CGN–nonmuscle myosin 2 (NM2) interaction is implicated in additional functions of CGN. For example, in three-dimensional (3D) culture, CGN is required for the formation of a single apical lumen of cysts of MDCK kidney epithelial cells (Mangan et al., 2016) and for the formation of isotropic colonies of Eph4 mammary epithelial cells (Yano et al., 2013). CGN interaction with microtubules and the Rab11-interacting protein FIP5 (RAB11FIP5) was implicated in the regulation of cyst morphogenesis (Mangan et al., 2016), but the possible role of CGN interaction with NM2 proteins was not investigated. Moreover, a variant within the C-terminal, NM2-binding region of CGN is associated with deafness in mice and humans (Zhu et al., 2023), but it is not clear whether this variant binds to NM2B and affects TJ membrane mechanics. Finally, CGN is a highly asymmetric homodimer comprising two globular heads and a 130 nm-long coiled-coil rod domain (Citi et al., 1988; Cordenonsi et al., 1999). The rod has been visualized in solution in either extended or folded conformation, depending on AMPK-dependent phosphorylation of serine and threonine residues in the head domain (Yano et al., 2018). However, nothing is known about the conformation of CGN at TJs and whether this is affected either by force or by binding to NM2B.

Here, we address these questions by analyzing the role of the NM2-binding region of CGN in the control of epithelial morphogenesis and in CGN nanoscale organization at TJ. We also examine the molecular interactions, expression and subcellular localization of the deafness (impaired hearing or IH) mutant and its impact on TJ membrane mechanics. The results indicate that the CGN–NM2 interaction is critical for CGN conformation and its role in morphogenesis and suggest that the IH variant causes deafness by impacting the CGN-dependent regulation of the mechanics of the membrane cortex of auditory epithelial cells.

## RESULTS

### The NM2B-binding region of CGN is required for normal epithelial morphogenesis

**Fig. 1.**
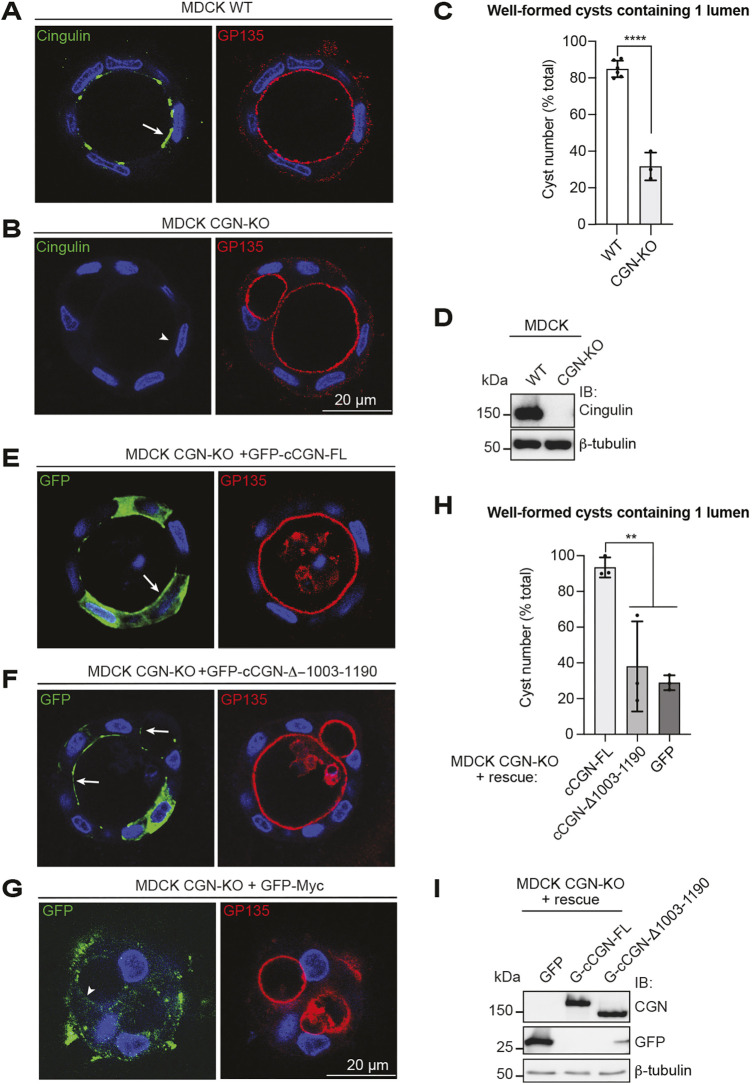
**The NM2B-binding region of CGN is required for normal morphogenesis of MDCK cysts in 3D culture.** (A,B) Immunofluorescence (IF) microscopy analysis of CGN (green) and GP135 (red, apical luminal marker) in WT (A) and CGN-KO (B) MDCK cysts. Scale bar: 20 µm. The arrow and arrowhead indicate normal and reduced/undetectable labeling for CGN, respectively. (C) Quantification of the percentage of cysts with one lumen in WT (white column) and CGN-KO (gray column) cells. Dots show replicates (*N*=3, *n*=60 cysts) and bars represent mean±s.d. Statistical significance of quantitative data was determined by one-way ANOVA test and the normality distribution was tested with Shapiro–Wilk test (****P<0.0001). (D) Immunoblot (IB) analysis of CGN in WT and CGN-KO MDCK cells, using β-tubulin as a loading control. Numbers on the left indicate migration of pre-stained molecular size markers. (E–G) IF microscopy analysis of GFP (green, GFP-tagged rescue constructs) and GP135 (red, apical luminal marker) in CGN-KO MDCK cells expressing either canine cCGN-FL (E), cCGN-Δ1003–1190 (F) or GFP alone (G). Arrows and arrowheads indicate junctional labeling of exogenous CGN constructs (either CGN-FL or CGN-Δ1003–1190) and undetectable junctional labeling for GFP alone, respectively. Scale bar: 20 µm. (H) Quantification of the percentage of cysts with one-lumen CGN-KO cells expressing WT full-length CGN (light gray column), C-terminally truncated CGN (cCGN-Δ1003–1190, gray column) or GFP (negative control, dark gray column). Statistical analysis was performed as in C. **P<0.001. (I) IB analysis of the expression of exogenous CGN constructs (either CGN-FL or CGN-Δ1003–1190) and the negative control construct GFP in CGN-KO rescued MDCK cells. Blots represent two independent experiments.

To examine the role of the CGN–NM2B interaction in epithelial morphogenesis, we analyzed by immunofluorescence microscopy the formation of cysts from wild-type (WT) and CGN-knockout (KO) MDCK cells cultured in 3D in Matrigel, and of CGN-KO cells rescued with different GFP-tagged CGN constructs. Antibodies against CGN, GFP and GP135 (also known as PODXL) were used to identify WT and CGN-KO cells, exogenous CGN, and the luminal apical borders, respectively. Consistent with previous results (Yano et al., 2013; Mangan et al., 2016), about 80% of cysts generated from WT cells had a single lumen (Fig. 1A, quantifications in Fig. 1C), whereas only about 30% cysts generated from CGN-KO cells had a single lumen (Fig. 1B, quantifications in Fig. 1C, immunoblot analysis of cell lines in Fig. 1D). CGN-KO cells rescued with WT CGN formed about 90% of one-lumen cysts (Fig. 1E, quantification in Fig. 1H), demonstrating the specificity of the phenotype. In contrast, cells expressing either the truncation mutant, which does not bind to NM2B, or GFP alone showed less than 40% one-lumen cysts (Fig. 1F,G, quantifications in Fig. 1H, immunoblot analysis of the lines in Fig. 1I). These results indicate that binding of CGN to NM2B is necessary to promote the formation of single-lumen cysts, i.e. normal epithelial morphogenesis.

### The IH CGN mutant binds to ZO-1 and is recruited to TJs, does not bind to NM2B, and impacts CGN-dependent mechanotransduction

The CGN variant associated with deafness (IH) consists of a deletion within exon 20 of the human gene, resulting in a frameshift mutation of 16 residues and a premature truncation of the C-terminal end of the coiled-coil rod domain and globular tail of CGN (Zhu et al., 2023). As this region of the coiled-coil rod sequence lies within the NM2B-binding region of CGN (residues 1015–1203 in human CGN) (Rouaud et al., 2023), we hypothesized that the IH mutant might not bind to NM2B. It was reported that this mutant is not targeted to junctions (Zhu et al., 2023). However, as CGN is recruited to TJs by ZO-1 and the ZO-1-binding region (D'Atri et al., 2002) is not affected by the variant, we also hypothesized that the IH mutant protein should interact with ZO-1 and be localized at junctions.

**Fig. 2.**
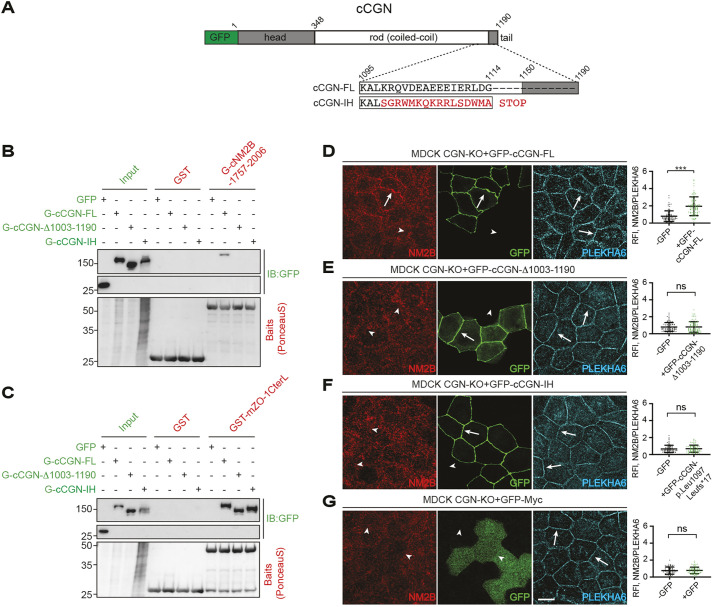
**The IH CGN mutant does not interact with NM2B but binds to ZO-1 and is recruited to tight junctions.** (A) Top: scheme of GFP-tagged canine CGN (cCGN-FL), with the GFP tag (green), globular head (gray), coiled-coil rod (white) and globular tail (gray) domains (amino-acid residue boundaries are indicated above the scheme). Bottom: C-terminal sequences of WT canine CGN (cCGN-FL, region 1095–1190 with specific residues indicated in the region 1095–1114) and corresponding sequences of the cCGN-IH mutant, with mutations identical to those of the human CGN impaired hearing (IH) variant. (B,C) IB analysis using anti-GFP antibodies of pulldowns using GST-tagged affinity-purified fragments of either the last 250 residues of cNM2B (CGN-binding region, GST-cNM2B 1757–2006) (B) or mouse ZO-1 (CGN-binding region, GST-mZO-1CterL; Rouaud et al., 2023) (C) as baits and either GFP-tagged full-length, truncated or IH-mutant forms of CGN expressed in HEK293T cells as preys (GFP–cCGN-FL, GFP–cCGN-Δ1003-1190 and GFP–cCGN-IH, respectively). Input lanes show normalized preys. Bottom panels show Ponceau Red-labeled baits. GST alone was the negative control bait and GFP–Myc was the negative control prey. Numbers on the left indicate migration of pre-stained markers. Blots represent two independent experiments. (D-G) IF microscopy analysis and localization (left) and quantification of junctional labeling (relative fluorescence intensity) (right) of NM2B in CGN-KO MDCK cells rescued with GFP–cCGN-FL (D), GFP–cCGN-Δ1003–1190 (E), GFP–cCGN-IH (F) or GFP–Myc alone (negative control, panel G). Arrows and arrowheads in D–G show normal and decreased/undetected junctional labeling, respectively. Quantifications of relative fluorescent intensity (RFI) shows the ratio between the junctional staining of NM2B versus the junctional marker PLEKHA6 (*n*=72 junctions) from two independent experiments. Data in quantifications are represented as mean±s.d. Statistical significance was determined by unpaired Mann–Whitney's test. ns, not significant; ***P≤0.001. Scale bar: 10 μm.

To test the hypotheses above, we designed a GFP-tagged mutant construct of canine CGN, with mutations identical to the human variant (Zhu et al., 2023) (Fig. 2A). Next, we used glutathione S-transferase (GST) pulldown assays to examine the ability of the IH mutant to interact with either NM2B or ZO-1 (Vasileva et al., 2022; Rouaud et al., 2023). Baits consisting of GST fused to the CGN-binding C-terminal fragments of either NM2B or ZO-1 were incubated with preys consisting of either full-length WT or IH-mutant CGN, expressed in HEK293T cells. A C-terminally truncated canine CGN (GFP–cCGN-Δ1003–1190) that does not bind to NM2B but binds to ZO-1 was used as additional control. Immunoblot analysis showed that the IH mutant (GFP–cCGN-IH) did not interact with the C-terminus of NM2B, similar to C-terminally truncated CGN (Fig. 2B), whereas WT full-length CGN (GFP–cCGN-FL) bound to NM2B (positive control; Rouaud et al., 2023) (Fig. 2B). Conversely, all constructs of CGN (WT, C-terminally truncated and IH mutant) interacted with the CGN-binding fragment of ZO-1 (Fig. 2C).

Next, we used immunofluorescence microscopy analysis to determine the cellular localization of the IH mutant and determine its ability to recruit NM2B to junctions in the context of CGN-KO cells. In CGN-KO cells, NM2B accumulation at junctions was significantly decreased and it was rescued by the expression of WT CGN (arrows in Fig. 2D), but not by expression of the C-terminal truncation of CGN lacking the NM2B-binding region (GFP–cCGN-Δ1003–1190, arrowheads in Fig. 2E) (Rouaud et al., 2023). When we rescued CGN-KO cells with the IH CGN mutant, the truncated protein was expressed at considerably lower levels with respect to the WT protein (Fig. S1), in agreement with previous observations (Zhu et al., 2023). However, at variance with previous observations (Zhu et al., 2023), the CGN IH mutant protein was normally localized at peripheral junctions, similarly to WT CGN (arrows, green channel, Fig. 2F). Junctional NM2B was not rescued by either the CGN IH mutant (arrowheads, red channel, Fig. 2F) or GFP alone (Fig. 2G, negative control). This was in agreement with the GST pulldown results, showing interaction of the IH mutant construct with ZO-1 but not with NM2B.

**Fig. 3.**
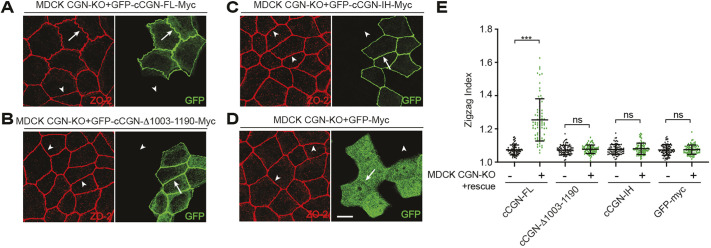
**The IH CGN mutant decreases tight junction membrane tortuosity.** (A–E) IF microscopy analysis (A–D) and measurement of the zig-zag index (E) for CGN-KO cells rescued with either full-length GFP-tagged canine CGN (GFP–cCGN-FL) (A), C-terminally truncated GFP-tagged cCGN (GFP–cCGN-Δ1003–1190) (B), C-terminally mutated GFP-tagged cCGN-IH (GFP–cCGN-IH) (C) or GFP–Myc alone (negative control) (D). Arrows and arrowheads show junctions with normal and decreased tortuosity, respectively. Scale bar: 10 μm. In E, ‘−’ and ‘+’ refer to the same cell line without and with exogenous expression of the rescue construct. Dots show replicates (*n*=80) and bars represent mean±s.d. Statistical significance was determined by unpaired Mann–Whitney's test. ns, not significant; ***P≤0.001.

To analyze the role of the IH mutant in the mechanotransduction function of CGN, we examined TJ membrane tortuosity in CGN-KO cells rescued with either WT or mutant CGN constructs (Fig. 3). As shown previously, WT CGN rescued TJ membrane tortuosity (arrows, Fig. 3A; quantifications in Fig. 3E), whereas the C-terminal deletion of CGN (CGN-Δ1003–1190) did not (arrowheads, Fig. 3B; quantifications in Fig. 3E) (Rouaud et al., 2023). The IH mutant did not rescue TJ membrane tortuosity, similar to the C-terminal deletion of CGN (CGN-Δ1003–1190) and to the negative control GFP (arrowheads in Fig. 3C,D; quantifications in Fig. 3E).

Taken together, these results demonstrate that the CGN IH mutant is normally targeted to TJs, consistent with its binding to ZO-1, whereas it does not bind to NM2B and does not recruit NM2B to junctions, impacting the CGN-mediated mechanoregulation of the plasma membrane cortex.

### The nanoscale organization of CGN at TJs is regulated by the NM2B-interacting region

**Fig. 4.**
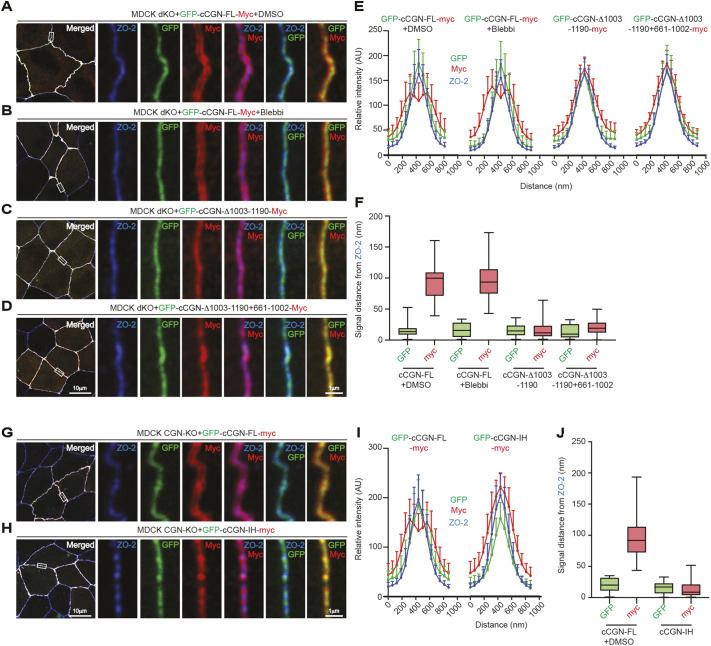
**The elongated conformation of CGN at tight junctions requires binding to NM2B but not myosin-generated force.** (A–D) IF microscopy analysis of the localization of the N-terminus (GFP, green) and C-terminus (Myc, red) of exogenous full-length CGN (A,B) and mutant (C,D) constructs, and of endogenous ZO-2 (blue), either in the presence of DMSO (panel A, negative control) or blebbistatin (panel B, Blebbi). The CGN mutants consisted of either deletion of the NM2B-binding region (cCGN-Δ1003–1190, panel C) or, in the same deletion, the addition of the sequence 661–1002 of CGN, which does not bind to NM2B (long CGN chimera, cCGN-Δ1003–1190+661–1002, panel D). Constructs were expressed in CGN and CGNL1 double-KO MDCK cells. High-magnification panels on the right correspond to highlighted white boxes in low-magnification micrographs on the left. (E,F) Line-scan analysis of signal distribution (E) and box plots of the distances of GFP and Myc signals from ZO-2 signals (F) (*n*=28 from two independent experiments), corresponding to the experiments shown in A–D. (G,H) IF microscopy analysis of the localization of the N-terminus (GFP, green) and C-terminus (Myc, red) of exogenous full-length CGN (G) and the IH-mutant construct (H), and of endogenous ZO-2 (blue). Constructs were expressed in CGN-KO MDCK cells. (I,J) Line-scan analysis of signal distribution (I) and box plots of the distances of GFP and Myc signals from ZO-2 signals (J) (*n*=28 from two independent experiments), corresponding to the experiments shown in G,H. Data in E,I are represented as mean±s.d. Boxes in F,J show the interquartile range, the whiskers show the range from the minimum to the lower quartile and from the upper quartile to the maximum, and the median is marked with a line. AU, arbitrary units. Scale bars: 10 μm (low magnification); 1 μm (high magnification).

Force and interaction with ZO-2 regulate the conformation of ZO-1, which can be either stretched or folded (Spadaro et al., 2017). Interestingly, CGN is an asymmetric molecule with a long coiled-coil domain, and folded and extended conformations of purified CGN have been described previously *in vitro* (Yano et al., 2018). However, the conformation of CGN in cells and whether this is affected by force or molecular interactions is not known. To address these questions, we expressed constructs of CGN with a N-terminal GFP tag and a C-terminal Myc tag in the background of CGN and paracingulin (CGNL1) double-KO MDCK cells and examined the localization of the tags on the two opposing sides of a bicellular TJ, using ZO-2 as a marker for the juxtamembrane region of TJs. In control cells expressing WT CGN and treated with solvent, the GFP signal, corresponding to the N-terminus of CGN, overlapped with the labeling for ZO-2 (Fig. 4A, green for GFP and blue for ZO-2), consistent with the N-terminus of CGN binding to the ZO-1–ZO-2 protein complex near the TJ membrane (Van Itallie and Anderson, 2014). In contrast, the Myc signals, corresponding to the two C-termini of CGN, on the two sides of the TJ membrane were detected as separated (Fig. 4A, red channel), with a distance of approximately 95 nm (mean±s.d.=94.4±28.8 nm, *n*=28, Fig. 4E,F) between the ZO-2 peaks and one of the Myc peaks at the C-termini of Myc-tagged CGN (Rouaud et al., 2023).

To test whether myosin-dependent force regulates the distance between the C-termini of CGN on the two sides of the TJ, we treated cells with blebbistatin, an inhibitor of myosin activity. Blebbistatin did not affect the distance between the ZO-2 peaks and one of the Myc peaks at the C-termini of Myc-tagged CGN (red channel, Fig. 4B; quantification in Fig. 4E,F), which was approximately 96 nm (96.5±28.3 nm, *n*=28, Fig. 4E,F), i.e. similar to untreated cells. In addition, treatment with blebbistatin did not affect the junctional accumulation of either endogenous CGN, NM2B, occludin (OCLN), ZO-2 and exogenous CGN in the context of either WT or double-KO (CGN-KO and CGNL1-KO) cells (Fig. S2). These observations indicate that NM2B accumulation at junctions does not depend on NM2-dependent activity and actomyosin force, but only on NM2B binding to CGN, and that the molecular architecture of TJ is stable following blebbistatin treatment. To test whether binding to NM2B, instead of force, regulates the nanoscale organization of CGN, we expressed the C-terminally truncated mutant, which does not interact with NM2B (GFP–cCGN-Δ1003–1190–Myc) (Rouaud et al., 2023). In this case, the two C-terminal tags of CGN on the two sides of the bicellular junction could not be spatially resolved (red channel, Fig. 4C; quantifications in Fig. 4E,F), suggesting that either CGN assumes a folded conformation or the C-termini are collapsed is a disorderly fashion in the juxtamembrane area. To exclude the possibility that the reduced distance between the N- and C-termini of CGN in the truncated mutants could be due to the shortening of the length of the coiled-coil rod, we generated a construct lacking the NM2B-binding site, but with the insertion of 341 residues corresponding to the region of the rod of CGN that does not interact with NM2B (long CGN chimera, GFP–cCGN-Δ1003–1190+661–1002, Fig. 4D; immunoblot analysis in Fig. S1). Despite this additional sequence, the C-termini of CGN on the two sides of the junctions could still not be resolved spatially (red channel, Fig. 4D; quantifications in Fig. 4E,F). This indicates that the conformation and/or nanoscale organization of CGN near the TJ membrane is affected by binding to NM2B, regardless of the physical length of the rod. As CGN also binds *in vitro* to NM2A, albeit with lower affinity, we cannot rule out that NM2A binding can also play a role.

Finally, we examined the IH mutant, by expressing either WT CGN or the IH mutant in the context of CGN-KO cells, which have the same phenotype as double CGN- and CGNL1-KO cells (Rouaud et al., 2023). The C-termini of WT CGN on opposing sides of the TJ membrane were separated (Fig. 4G; quantifications in Fig. 4I,J). Instead, consistent with the fact that the IH mutant does not bind to NM2B (Fig. 2), the C-termini of the IH-mutant CGN molecules on opposing sides could not be resolved (Fig. 4H; quantifications in Fig. 4I,J).

The loss of NM2B binding to CGN prevents the formation of the ZO-1–CGN–NM2B complex (Rouaud et al., 2023). To test the ability of the new mutant constructs to mediate the formation of the trimolecular complex, we carried out GST pulldown experiments using either the CGN-binding region of ZO-1 or the CGN-binding C-terminal region of NM2B as a bait, and either WT or mutant CGN constructs as preys (Fig. S3). Both WT CGN and CGN lacking the NM2B binding region interacted with the ZU5-containing fragment of ZO-1 (Fig. S3A) (Rouaud et al., 2023). However, when NM2B was used as a bait, all the CGN constructs lacking the NM2B-binding region (prey quantifications in Fig. S3B,D; GST pulldowns in Fig. S3C,E), including the long chimera (Fig. S3B,C) and the IH mutant (Fig. S3D,E), failed to form the ZO-1–CGN–NM2B trimolecular complex owing to the lack of CGN–NM2B interaction. Conversely, WT CGN could bind to both NM2B and ZO-1 and thus form the trimolecular complex (Fig. S3C,E).

In summary, the NM2-binding region of CGN is required to maintain the CGN C-terminus away from the TJ and promote the formation of a trimolecular NM2B–CGN–ZO-1 complex, suggesting that binding of CGN to both ZO-1 at its N-terminus and NM2B at its C-terminus regulates the nanoscale organization of CGN at TJs.

## DISCUSSION

How specific molecules contribute to the architectural organization of the contractile actomyosin cytoskeleton, to epithelial morphogenesis and to force transduction in physiological and pathological contexts are fundamental questions in cell biology. Here, based on our recent discovery that the TJ protein CGN recruits NM2B to TJs to regulate TJs and apical membrane mechanics (Rouaud et al., 2023), we examined the role of the CGN–NM2B interaction in epithelial morphogenesis and in the nanoscale organization of CGN at TJs. We also analyzed the biochemical interactions, cellular localization and role of a CGN IH variant that causes deafness in TJ membrane mechanics. In humans, *de novo* lumen formation is an important mechanism of epithelial tissue morphogenesis, and it involves the actomyosin cytoskeleton, polarity complexes and polarized membrane traffic (Bryant et al., 2010; Blasky et al., 2015). The following model of apical lumen formation *in vitro* postulates a key role for the apical membrane initiation site (AMIS), a transient structure that contains CGN and apical endosomes, and matures into a TJ as the lumen forms (Li et al., 2014; Mangan et al., 2016). CGN is required for apical lumen formation (Mangan et al., 2016), and it was proposed that CGN acts by binding to the Rab11-binding protein FIP5, thus targeting Rab11–FIP5 endocytic vesicles to the AMIS (Mangan et al., 2016). CGN binding to microtubules was also proposed to play a role in AMIS formation (Mangan et al., 2016). However, our WT and mutant constructs had no mutation in the globular head domain of CGN, which contains the sequences involved in interaction with microtubules (Yano et al., 2013; Mangan et al., 2016; Yano et al., 2018). Moreover, although we did not examine the localization of FIP5 in cysts of CGN-KO MDCK cells expressing our WT and mutant constructs, FIP5 binds to the Rod1 region of the coiled-coil CGN rod (Mangan et al., 2016), which is distinct from the Rod2 region, which binds to NM2 proteins (Rouaud et al., 2023). MgcRacGAP and GEF-H1 also bind to the Rod1 region, and they are normally recruited to junctions when the C-terminally truncated CGN mutant is expressed in the context of CGN-KO cells (Rouaud et al., 2023). Thus, our results suggest a mechanism whereby the role of CGN in lumen formation depends primarily on the interaction of the CGN C-terminus with NM2B, rather than on interactions with the microtubule or participation in endocytic traffic. However, as the Rod2 domain of CGN also binds *in vitro* to NM2A, albeit with lower affinity (Cordenonsi et al., 1999; Rouaud et al., 2023), and as we cannot exclude interactions with additional ligands (Rouaud et al., 2023), a role for NM2A or other ligands cannot be formally excluded. How the CGN–NM2B interaction regulates lumen formation is unclear. Rac1-dependent branched actin polymerization is important in AMIS formation (Mangan et al., 2016), and CGN controls the cortical anchoring of γ-actin (Rouaud et al., 2023), which has been involved in the formation of a dense submembrane lamellipodial actin network (Dugina et al., 2022). As γ-actin has unique viscoelastic properties (Nietmann et al., 2023), CGN might act by regulating the precise spatial organization of specific actin and myosin isoforms and thus affect the tethering of the ZO-1-associated complexes to the contractile actomyosin bundle that remodels the apical membrane during lumen formation (Varadarajan et al., 2019). Another potential mechanism is linked to the role of CGN in regulating Rho GTPases. Depletion of CGN results in a GEF-H1-dependent increase in RhoA activity and reorganization of the actin cytoskeleton, including increased basal stress fibers (Aijaz et al., 2005; Guillemot and Citi, 2006; Mangan et al., 2016). However, we found no evidence for increased RhoA activity either in our CGN-KO cultured cells or in CGN-KO mouse epithelia (Guillemot et al., 2012; Vasileva et al., 2022; Rouaud et al., 2023). Moreover, the multiple-lumen phenotype of CGN-KO cells is not rescued by constructs that recruit GEF-H1, but not NM2B, to TJs (Rouaud et al., 2023). Thus, we conclude that, in our experimental model, the role of CGN can more likely be explained by its regulation of actin and myosin through binding of NM2B, rather than its regulation of Rho GTPases. It is unclear whether CGN regulates epithelial morphogenesis *in vivo*. CGN-KO embryoid bodies and epithelial tissues of CGN-KO mice develop normally and do not show altered actomyosin organization (Guillemot et al., 2004, 2012). This could be due to redundant functions of CGN and CGNL1, as CGNL1 controls cyst morphogenesis in mCCD cells, in which CGNL1 is abundantly expressed (Flinois et al., 2024). By binding to NM2 proteins (Rouaud et al., 2023), CGNL1 could therefore compensate for the lack of CGN in CGN-KO mouse epithelia but not in MDCK cells, in which CGNL1 expression is low (Vasileva et al., 2017; Rouaud et al., 2023). In summary, our data suggest that the tethering of NM2B by CGN to TJs plays an important role in cyst morphogenesis in MDCK cells.

A second goal of this study was to analyze the CGN truncation mutation [c.3330delG (p.Leu1110Leufs*17)] that causes autosomal dominant non-syndromic hearing loss (Zhu et al., 2023). CGN is enriched in the cuticular plate, a specialization of the apical surface of auditory cells of the organ of Corti, which is essential for hearing function (Du et al., 2019). Both the IH mutant and the KO of CGN were reported to affect the morphology of the cuticular plate and hair bundle and induce the degeneration of outer hair epithelial cells after exposure to high frequency sound, leading to deafness (Zhu et al., 2023). The truncation entails the loss of a fragment of 95 residues within the Rod2 domain (corresponding to residues 1095–1190 in canine CGN). This region is about half the length of the minimal fragment that we identified previously as NM2B-binding site (188 residues, residues 1015–1203 of human CGN; Rouaud et al., 2023). Thus, by showing that the IH mutant abolishes interaction of NM2B with CGN, we were able to narrow down more precisely the residues required for NM2B-binding. At variance with the reported cytoplasmic localization of the IH mutant when exogenously expressed in MDCK cells (Zhu et al., 2023), our results show that the IH mutant is correctly localized at TJs, consistent with its interaction with ZO-1. This discrepancy might be due to different culture conditions, monolayer confluency, transfection and fixation protocols, and levels of transgene expression. More importantly, we show that the IH mutant affects CGN-mediated mechanical regulation of the TJ membrane, consistent with its loss of binding to NM2B, which produces the same phenotype (Rouaud et al., 2023). Although we did not measure apical membrane stiffness in CGN-KO cells expressing the IH mutant, the phenotypes of TJ membrane tortuosity and apical membrane stiffness are tightly correlated (Rouaud et al., 2023). Thus, it is safe to predict that CGN-KO MDCK cells expressing the IH mutant would show decreased apical membrane stiffness. Moreover, considering that the CGN IH mutant is not well expressed in cells (Zhu et al., 2023), the mutation effectively phenocopies a KO of CGN, which results in both mechanical phenotypes (Rouaud et al., 2023). Future experiments should measure the apical stiffness of hair cells lacking CGN. Based on our observations, we propose that deafness and hair cell degeneration is due to altered mechanoregulation of the apical and TJ membranes caused by the loss of CGN function. CGN could act by affecting the localization of either NM2B or γ-actin (Rouaud et al., 2023) or both, as variants of γ-actin have also been linked to hereditary deafness (Zhu et al., 2003). Additional studies will be required to test these hypotheses. The notion that actomyosin organization is critical for the morphology and function of the mechanotransduction apparatus of hair cells and the cuticular plate is supported by studies on LMO7, a protein that controls the density and organization of actin filaments in auditory hair cells (Du et al., 2019). Similarly to CGN, LMO7 is enriched in both cuticular plate and cell–cell junctions, and the KO of LMO7 leads to defects in the cuticular plate and stereocilia, loss of outer hair cells and hearing loss (Du et al., 2019). Thus, multiple junctional and/or cytoskeletal components have non-redundant roles in auditory function through the fine control of the organization and mechanoresponses of the actomyosin cytoskeleton.

A final goal of this study was to explore the relevance of the CGN–NM2B interaction and force in CGN conformation and nanoscale organization at TJs. CGN is interposed between the C-terminus of ZO-1, which binds to the N-terminus of CGN and the NM2-containing actomyosin cytoskeleton (Rouaud et al., 2023). Our finding that blebbistatin treatment does not affect the positioning of the C-terminus of CGN with respect to the TJ membrane suggests that abolishing myosin-dependent force is not sufficient to alter CGN conformation and/or nanoscale organization at TJs. Conversely, in the absence of the NM2-interacting region, the C-terminus of CGN collapses towards the TJ membrane, indicating an important role of NM2 interaction in maintaining CGN conformation at the TJ. Previous work highlighted the role of microtubule binding and phosphorylation by AMPK in regulating the conformation of purified CGN *in vitro* (Yano et al., 2013; Mangan et al., 2016; Yano et al., 2018). However, our constructs had no mutation in the globular head region of CGN, which contains microtubule-binding sequences and AMPK phosphorylation sites, suggesting that, within cells, the head domain of CGN does not contribute to the regulation of conformation, beyond its role in tethering to ZO-1. These and other observations (Spadaro et al., 2017; Vasileva et al., 2022; Nguyen et al., 2024) show that the conformation and nanoscale organization of junctional proteins within cells is controlled redundantly by force and by multiple molecular interactions with cytoskeletal and junctional proteins.

In summary, here, we provide evidence that the NM2-binding region of CGN is important in epithelial morphogenesis and CGN nanoscale organization at TJ, and we suggest that CGN involvement in the pathophysiology of hearing depends on its role as an organizer of the actomyosin cytoskeleton to regulate the mechanics of apical and junctional membrane cortexes. This advances our knowledge on the molecular basis of CGN functions and architectural organization, and its implication in disease.

## MATERIALS AND METHODS

### Experimental models and transfection

MDCK (Madin–Darby canine kidney II cell line, female; CGN WT, CGN-KO, and CGN and CGNL1 double-KO) and HEK293T cells were cultured at 37°C at 5% CO_2_ in Dulbecco's modified Eagle medium (DMEM; P04-04500, PAN Biotech) supplemented with 10% heat-inactivated fetal bovine serum (FBS, PAN Biotech, Aidenbach, Germany) and 1% non-essential amino acids (MEM NEAA, PAN Biotech) (Rouaud et al., 2023). CGN-KO cell lines stably rescued with either CGN-FL, CGN-Δ1003–1190 or GFP alone were generated by transfection, sorting and screening (Rouaud et al., 2023). Blebbistatin (B0560, Sigma-Aldrich) treatment was 50 μM for 4 h and DMSO was used as control solvent. For transfections (rescue experiments), cells grown on glass coverslips in 24-well plates were seeded at a density of (1–2)×10^5^ cells/well, transfected the next day using jetOPTIMUS DNA transfection reagent (117-15, PolyPlus) according to the manufacturer's protocol and fixed for immunofluorescence at 3 days post transfection. HEK293T cells were plated in 10 cm dishes (2×10^6^ cells/dish), transfected the next day using jetOPTIMUS DNA transfection reagent and lysed 72 h post transfection. All cell lines were routinely tested for contamination by mycoplasma using PCR, and their source is indicated in Table S1.

### Antibodies and plasmids

The following primary antibodies against the indicated proteins were used for either immunoblotting (IB) or immunofluorescence (IF) microscopy at the indicated dilutions: mouse anti-cingulin (22BD5A1, Citilab; IF: 1:500); rabbit anti-NM2B (909901, Biolegend; IF: 1:250); rat anti-PLEKHA6 (RtSZR127, Sluysmans et al., 2021; IF: 1:100); mouse anti-GFP (11814460001, Roche; IF: 1:200, IB: 1:1000); goat anti-ZO-2 (sc-8148, Santa Cruz Biotechnology; IF: 1:100); rabbit anti-Myc (06-549, Millipore; IF: 1:200); rabbit anti-HA (sc-805, Santa Cruz Biotechnology; IB: 1:1000); and mouse anti-GP135 (3F2/D8, BD Biosciences; IF: 1:5).

The secondary antibodies for immunoblotting (Dako) were polyclonal HRP-conjugated goat anti-mouse (P0447) and anti-rabbit (P0448) antibodies and were diluted at 1:3000. Antibody validation is described in Sluysmans et al. (2021) and Rouaud et al. (2023).

For immunofluorescence, the following secondary antibodies (Jackson ImmunoResearch) were used at a dilution of 1:300: anti-mouse-IgG (715-546-151) conjugated to Alexa Fluor 488; anti-rabbit-IgG (711-165-152) conjugated to Cy3; anti-rat-IgG (712-175-153) conjugated to Cy5; and anti-goat-IgG (705-606-147) conjugated to Alexa Fluor 647. Additional details on antibodies (catalog numbers, RRIDs, etc.), plasmids and reagents for this study are listed in Table S1.

### Immunofluorescence

For immunofluorescence analysis, MDCK cells were seeded on 12-mm #1.5 round glass coverslips in 24-well plates at a density of (1–2)×10^5^ cells/well. 72 h after cells were fixed in 1% paraformaldehyde for 12 min, followed by two rinses with PBS and incubation with methanol at −20°C for 5 min, followed by gradual rehydration in PBS (Rouaud et al., 2023). Cells were permeabilized with 0.2% Triton X-100 [5 min at room temperature (RT)] and saturated 20 min with 2% BSA in PBS.

Incubation with primary antibodies was carried out for 2 h at RT, followed by three washes with PBS, incubation with secondary antibodies (1 h at RT) and washing. Coverslips were mounted with Fluoromount-G (0100-01, SouthernBiotech).

Slides were imaged on a Zeiss LSM800 confocal microscope using a Plan-Apochromat 63×/1.40 oil objective or a Plan-Apochromat 100×/1.40 oil objective (1024×1024 pixels). Maximum-intensity projections of *z*-stack images (typically three to six confocal planes over 0.9–1.8 µm, step size=0.3 µm) were obtained. Images were extracted from .lif, .lsm or .czi files using ImageJ, adjusted and cropped using Adobe Photoshop, and assembled in Adobe Illustrator figures.

### GST pulldown and immunoblotting

For GST pulldowns, GST-tagged protein baits were expressed in BL21 bacteria and purified by affinity chromatography on magnetic beads as described in Sluysmans et al. (2021). Preys were tagged (either GFP or HA) full-length and mutant proteins expressed in HEK293T cells. Pulldowns were carried out as described in Sluysmans et al. (2021).

Samples were mixed with SDS sample buffer, incubated at 95°C for 5 min, and subjected to SDS-PAGE (9% acrylamide). For immunoblotting, gels were transferred onto nitrocellulose membranes (0.45 μm) (85 V for 2 h at 4°C). Then, nitrocellulose sheets were blocked in TBS containing milk (150 mM NaCl, 50 mM Tris-HCl pH 7.5, 0.1% Tween 20, 5% skimmed milk) for 1 h and incubated with primary antibody overnight at 4°C, followed by washing, incubation with secondary HRP-labeled antibody (1 h at RT), washing, and development of ECL luminescence reaction (Amersham ECL, Advansta, San Jose, CA, USA). Numbers on the left of immunoblots indicate migration of pre-stained molecular size markers (kDa). Uncropped blots (either complete or portions, due to utilization of one membrane for multiple blots) are shown in Figs S4–S7.

### Growth of MDCK cells in 3D culture

To induce 3D cyst morphogenesis, 40 µl of Matrigel (BD Biosciences, 354230) was deposited on a glass coverslip placed into a 24-well plate and allowed to solidify for 30 min at 37°C. In parallel, cells were trypsinized, resuspended in DMEM, centrifuged at 1500 ***g*** for 3 min, and resuspended in 2 ml of S-MEM (Gibco, 11380-037) to allow a single-cell suspension. 3.5×10^4^ cells from the resulting suspension were mixed in a 1:1 ratio with a solution composed of 2× standard DMEM, 4% Matrigel and 10 ng/ml epidermal growth factor (E5036, Sigma-Aldrich). Subsequently, 400 µl of this mixed solution was plated into the coverslip containing solidified Matrigel. On day 7, the cysts were fixed with a 1:1 mix of methanol and acetone for 11 min at −20°C and permeabilized with PBS containing 0.5% Triton X-100 for 10 min at RT. After washing with PBS containing 100 mM glycine, cysts were blocked with IF buffer (PBS containing 100 mM glycine, 0.5% BSA, 0.2% Triton X-100 and 0.05% Tween 20) for 2 h at RT. Incubation with primary antibodies was carried out at RT overnight, followed by three washes with IF buffer. Then, cysts were incubated with DAPI and secondary antibodies (1:300) for 4 h at RT, followed by three washes with IF buffer. Coverslips containing cysts wrapped in Matrigel were mounted with Fluoromount-G (Debnath et al., 2003). Slides were imaged on a Zeiss LSM800 confocal microscope using a Plan-Apochromat 63×/1.40 oil objective at a resolution of 1024×1024 pixels. Images were extracted from .czi files using ImageJ, adjusted, cropped and assembled using Affinity Designer.

### Quantification and statistical analysis

Data processing and analysis were performed in GraphPad Prism 8. All experiments were carried out at least in duplicate, and data are shown either as dot plots, histograms or line graphs (with mean and standard deviation indicated). Statistical significance was determined by unpaired Mann–Whitney's test (when comparing two sets of data), Kruskal–Wallis test followed by Dunn's multiple comparison, or one-way ANOVA with the normality distribution tested with Shapiro–Wilk test, as detailed in the figure legends (ns, not significant; *P≤0.5; **P≤0.01; ***P≤0.001; **** *P*≤0.0001). For quantification of cyst morphogenesis, the proportion of cysts forming one, two or multiple lumens were counted. 60 cysts were analyzed.

### Analysis of immunofluorescence data

For the quantification of junctional immunofluorescence signal, pixel intensity for each channel was measured in the selected junctional area using the polyhedral tool of ImageJ, and the averaged background signal of the image was subtracted. Relative intensity signal was expressed as a ratio between the signal of the protein of interest and an internal junctional reference (PLEKHA6). Typically, 50–70 junctional segments were analyzed for each of the independent duplicate or triplicate experiments.

For the analysis of distances of TJ proteins and their N- and C-termini, slides were imaged on a Zeiss LSM800 confocal microscope using a Plan-Apochromat 100×/1.40 oil objective at a resolution of 1024×1024 pixels. Linescan Analysis (ImageJ) was carried out on a 1 µm linear distance across the junction, centering on the maximum-intensity signal. Using the Plot Profile plugin of ImageJ, pixel intensities (*y*-axis) of red, green and far-red fluorophores were plotted as a function of distance (*x*-axis) across the junction. The *x*-coordinate of the maximum intensity peak was determined using the mean function of the Gaussian curve (Prism software).

For the measurement of the zig-zag index (L_TJ_/L_St_, ratio between the actual length of bicellular junction and the distance between two vertexes), we used the method described in Tokuda et al. (2014), and measured the length of the TJ (L_TJ_) by using the freehand line trace in ImageJ, and the straight length of junction (L_St_) by using a straight line between vertexes. Typically, between 60 and 80 intercellular junctions were analyzed.

## Supplementary Material

10.1242/joces.262353_sup1 Supplementary information
