# Supplementary material for: Cingulin–nonmuscle myosin interaction plays a role in epithelial morphogenesis and cingulin nanoscale organization
Source: J Cell Sci. 2024 Sep 25;137(18):jcs262353. doi: 10.1242/jcs.262353 (PMC11449440; doi:10.1242/jcs.262353)
Supplement: Supplementary information [file joces-137-262353-s1.pdf]

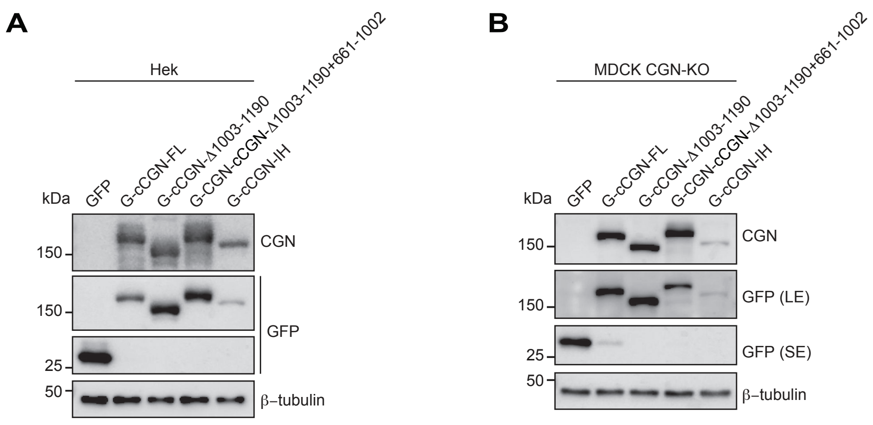

Fig. S1. CGN expression in HEK cells and rescued MDCK cells.

(A-B) IB analysis of the expression levels of exogenous full-length CGN (cCGN-FL), C-terminally truncated CGN (cCGN-Δ1003-1190), long CGN chimera (cCGN-Δ1003-1190+661-1002), IH mutant of CGN (cCGN-IH) and GFP-Myc (negative control) either in HEK cells (A) or in CGN-KO (rescued) MDCK cells (B). β-tubulin was used as a loading control. (SE) and (LE) in (B) indicate short and long exposure, respectively.

Journal of Cell Science • Supplementary information

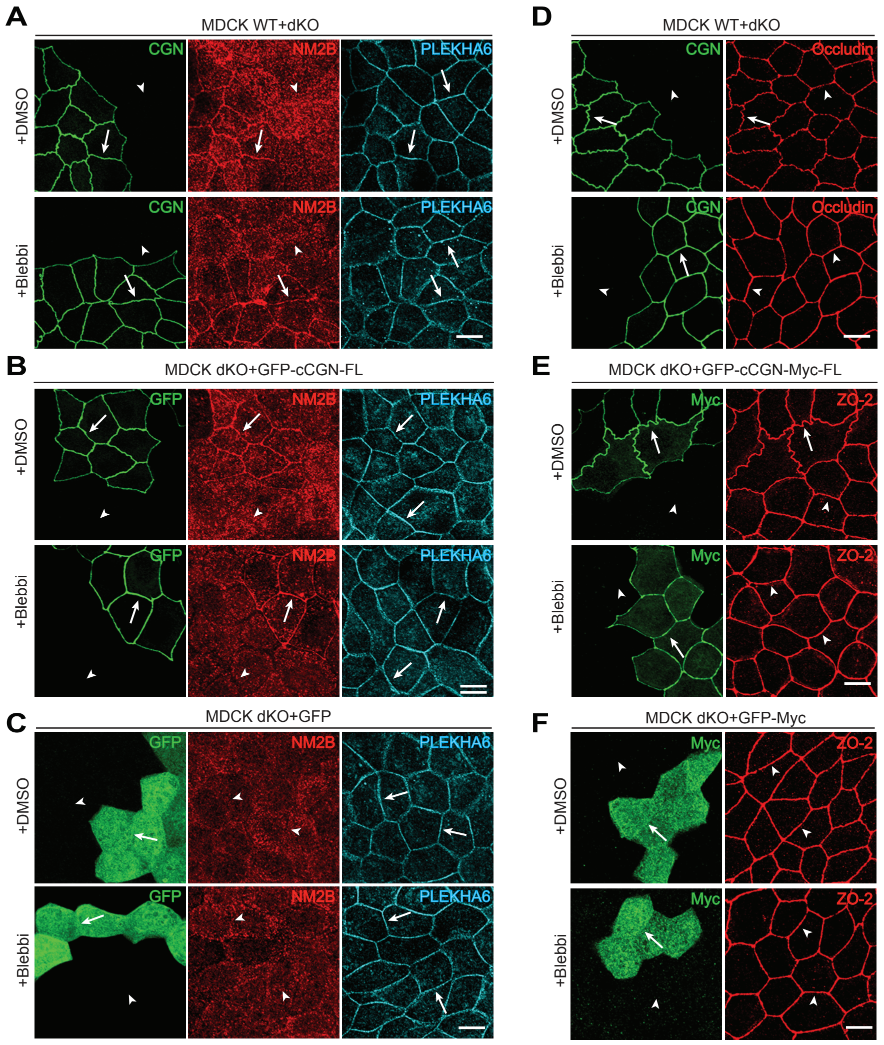

Fig. S2. Blebbistatin reduces TJ membrane tortuosity without affecting NM2B localization.

(A) IF microscopy analysis of NM2B in mixed cultures of WT + CGN-CGNL1 double-KO (dKO) MDCK cells without (to panel) or with Blebbistatin treatment (bottom panel).

(B-C) IF microscopy analysis of the localization of endogenous NM2B in dKO-MDCK cells, upon expression of GFP-tagged constructs of full-length (FL) CGN with (bottom panel) or without Blebbistatin (top panel) (B), or GFP alone with (bottom panel) or without Blebbistatin (C). Arrows and arrowheads show normal and decreased/undetected junctional labeling, respectively. Scale bars= 10 μm.

(D-F) IF microscopy analysis of ZI in mixed cultures of WT + CGN-CGNL1 double-KO (dKO) MDCK cells with (bottom panel) or without Blebbistatin treatment (4h, 50μM, top panel) (D) and in dKO-MDCK cells, upon expression of GFP-tagged constructs of full-length (FL) CGN with (bottom panel) or without Blebbistatin (top panel) (E) or GFP alone with (bottom panel) or without Blebbistatin (top panel) (F). Arrows and arrowheads show tortuosity and decreased/undetected tortuosity, respectively. Scale bars= 10 μm.

Journal of Cell Science • Supplementary information

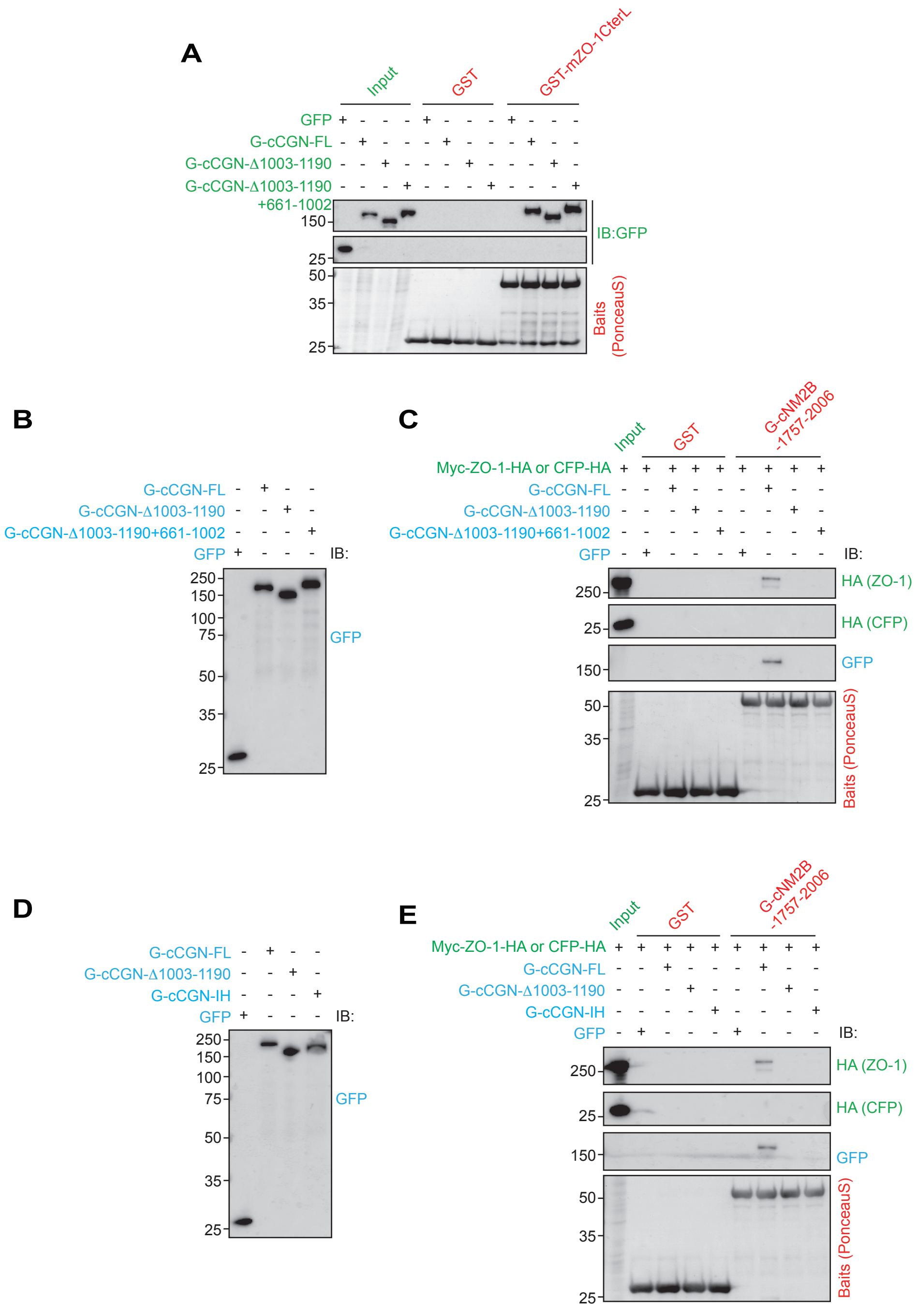

Journal of Cell Science • Supplementary information

Fig. S3. The effect of CGN mutations on the formation of the ZO-1-CGN-NM2B trimolecular complex.

(A) IB analysis, using anti-GFP antibodies, of pulldowns using affinity purified GST-tagged fragments of mouse ZO-1 C-terminal fragment (GST-mZO-1CterL) as baits and the following constructs as preys: GFP-tagged full-length of CGN (cCGN-FL); CGN mutant with a deletion of the NM2B-binding region (cCGN-Δ1003-1190); CGN mutant with the same deletion plus the addition of sequences 661-1002 of CGN, which do not bind to NM2B (long CGN chimera, cCGN-Δ1003-1190 +661-1002); GFP alone (negative control). Preys were expressed in HEK cells.

(B, D) IB analysis, using anti-GFP antibodies of normalization of third protein preys (blue) used in tri-molecular GST pulldowns (C and E): exogenous full-length CGN (cCGN-FL); C-terminally truncated CGN (cCGN-Δ1003-1190); long CGN chimera (cCGN-Δ1003-1190+661-1002); IH mutant of CGN (cCGN-IH); GFP-Myc (negative control).

(C, E) IB analysis, using either anti-HA or anti-GFP antibodies, of tri-molecular GST pulldowns. Either a C-terminal fragment of mouse ZO-1 (GST-mZO-1CterL) or GST alone (negative control) were used as baits (red). Either Myc-ZO-1-HA or CFP-HA (negative control) were used as preys (green, identified by anti-HA). The third proteins (blue, normalized in B and D, identified by anti-GFP) were either GFP (negative control), or GFP-tagged full length CGN, or C-terminally truncated CGN, or long chimera of CGN, or impaired-hearing mutant of CGN. Bottom panels in A, C and E show Ponceau-red labeled baits. Numbers on the left indicate migration of pre-stained markers.

Journal of Cell Science • Supplementary information

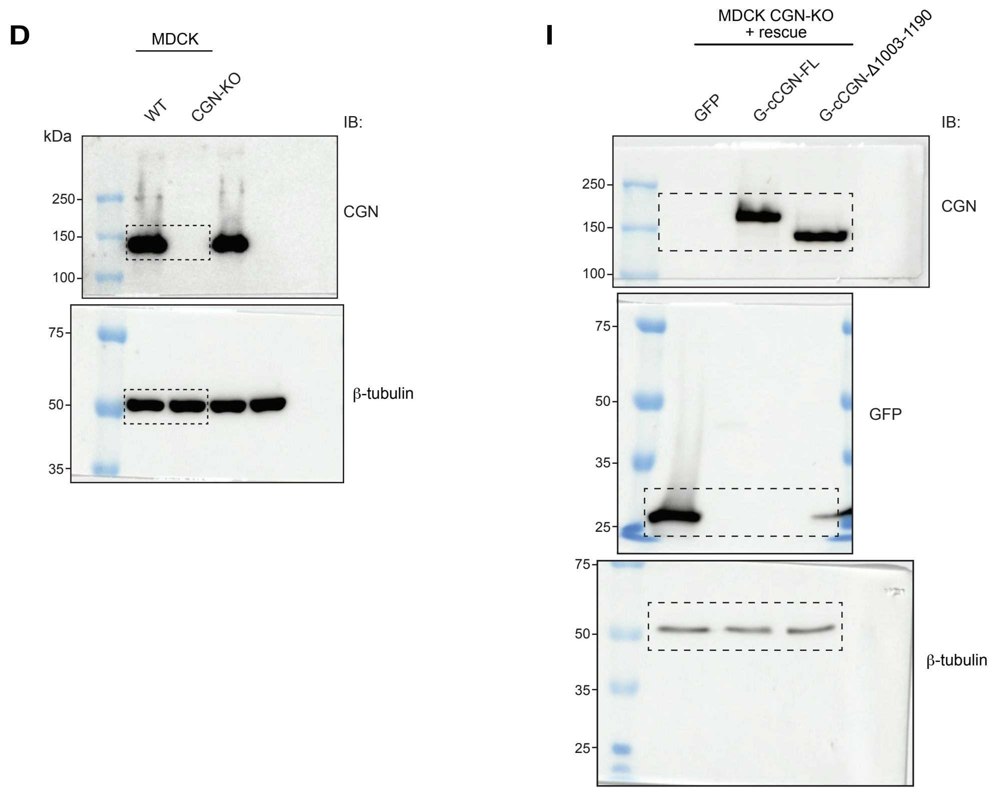

Fig. S4. Blot transparency in Figure 1D and 1I.

The area used in Figure 1D and 1I of this study is highlighted with a dashed line box.

Journal of Cell Science • Supplementary information

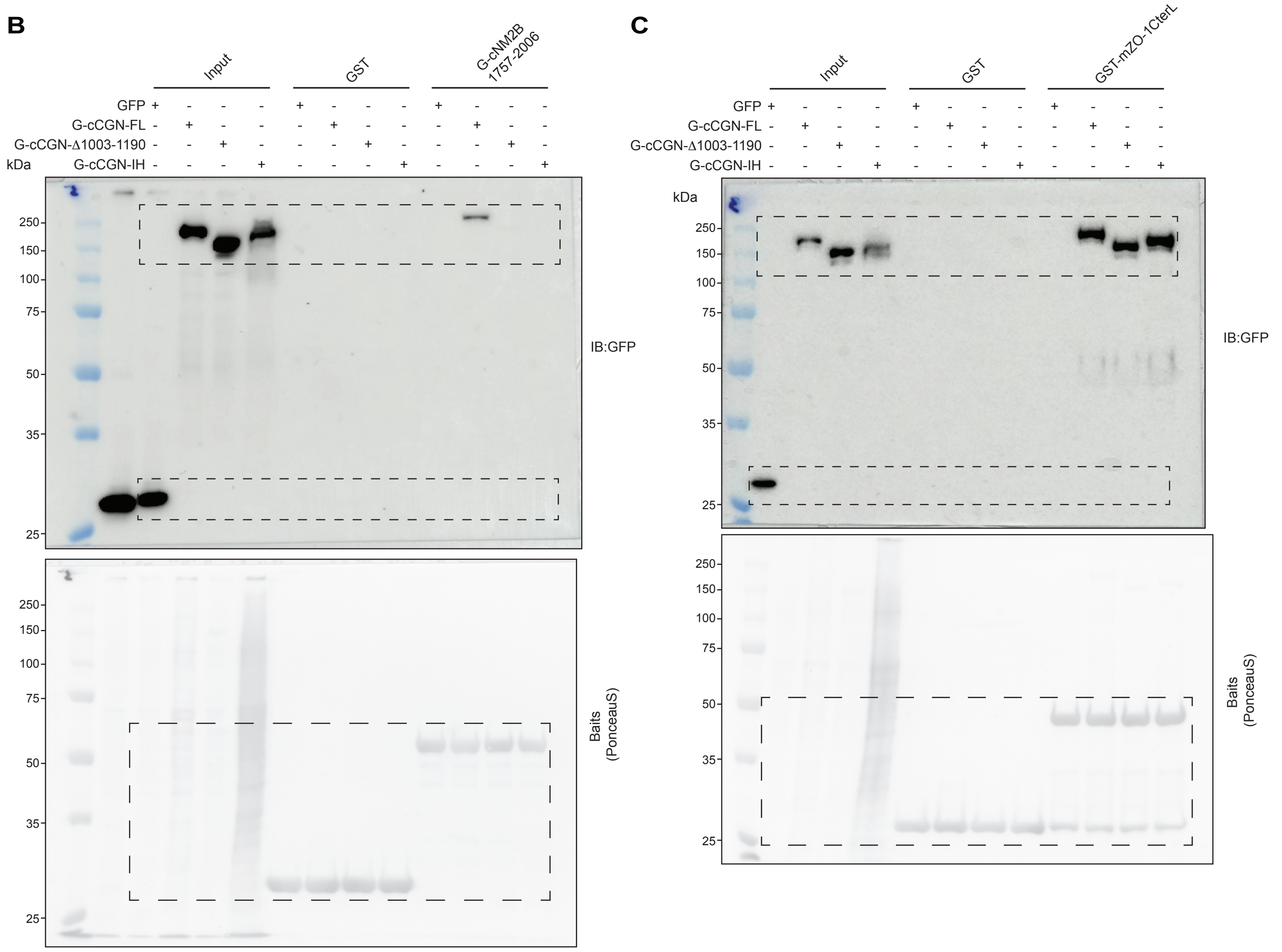

Fig. S5. Blot transparency in Figure 2B-C.

The area used in Figure 2B-C of this study is highlighted with a dashed line box.

Journal of Cell Science • Supplementary information

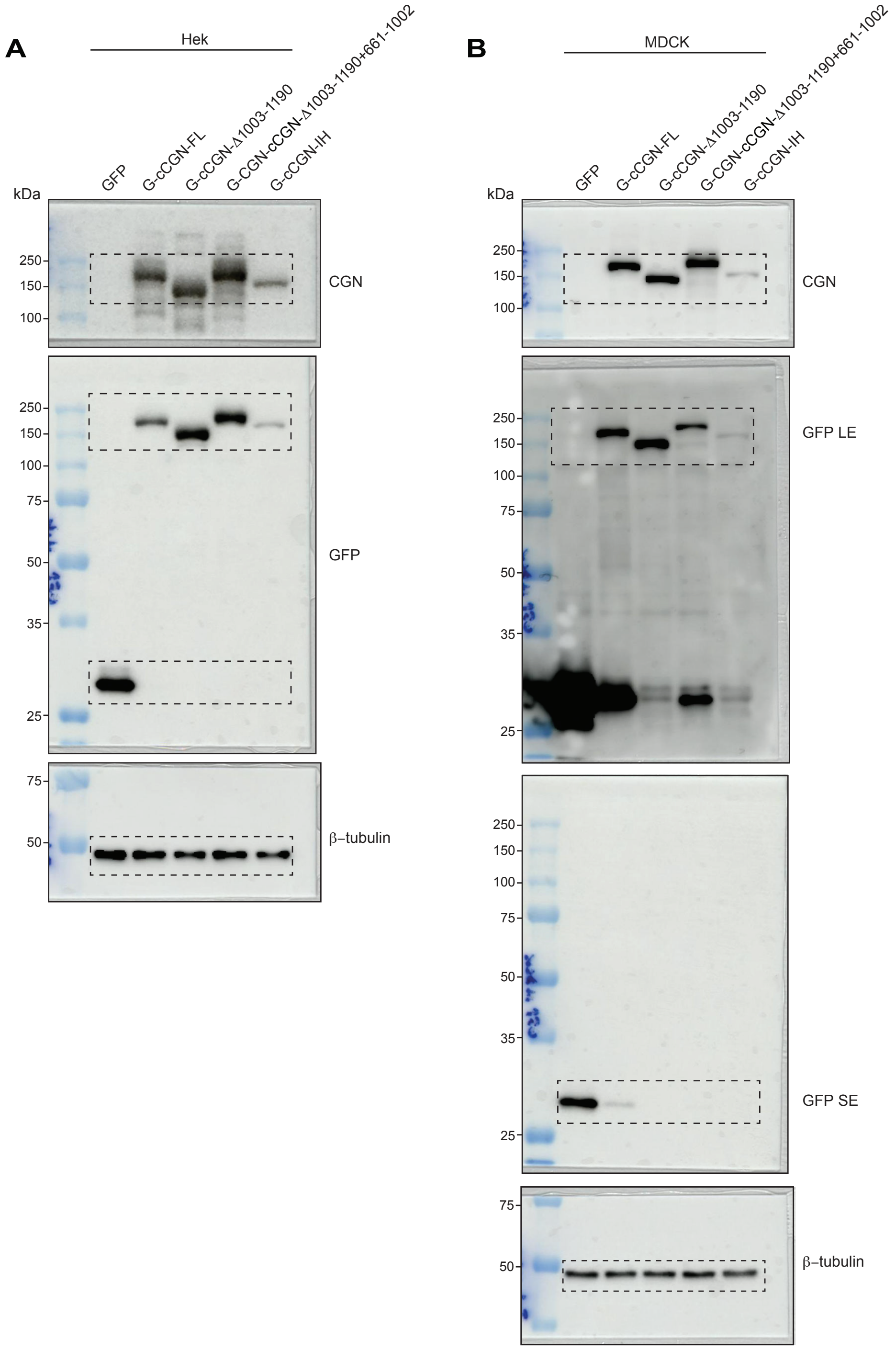

Fig. S6. Blot transparency in Figure S1A-B.

The area used in Figure S1A-B of this study is highlighted with a dashed line box.

Journal of Cell Science • Supplementary information

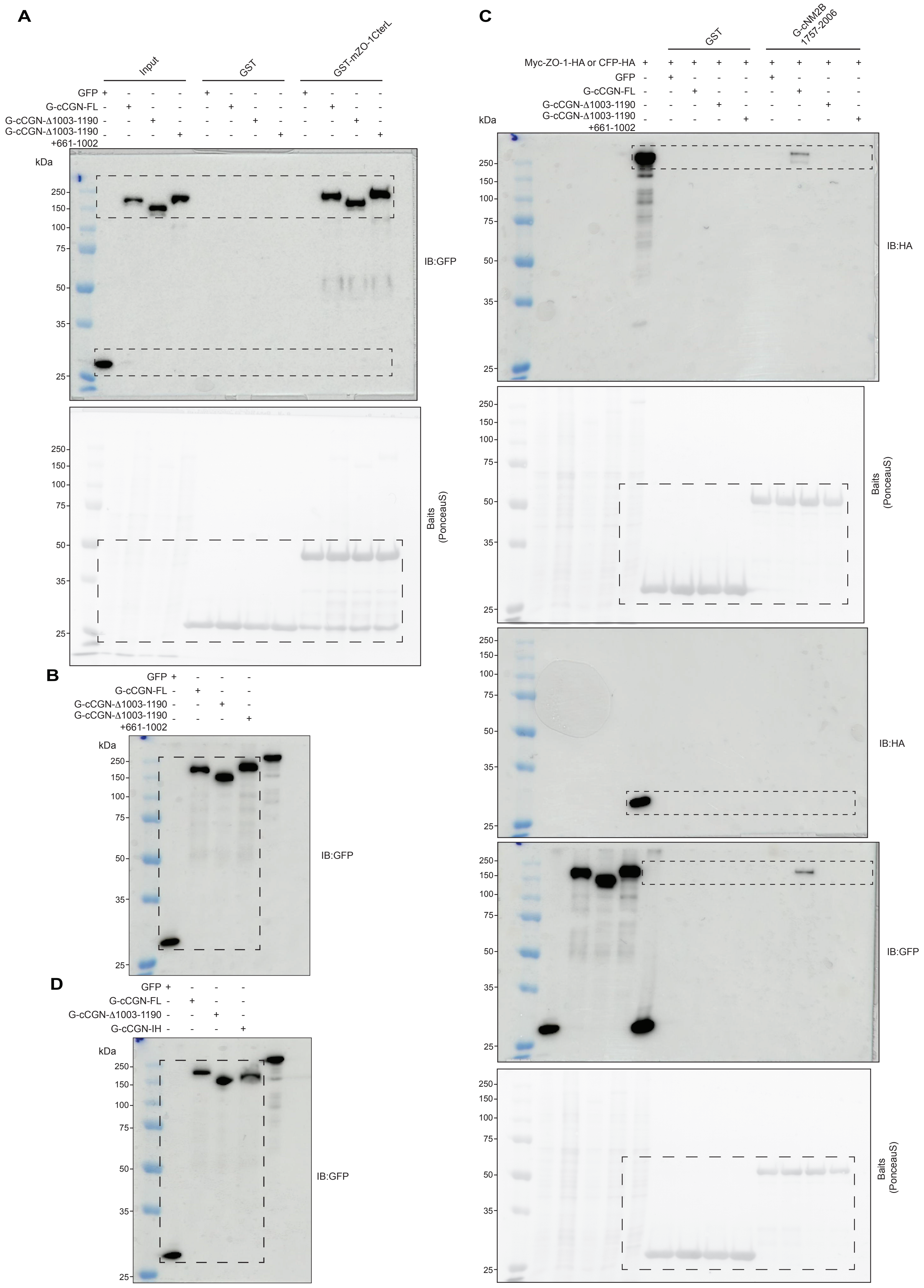

Journal of Cell Science • Supplementary information

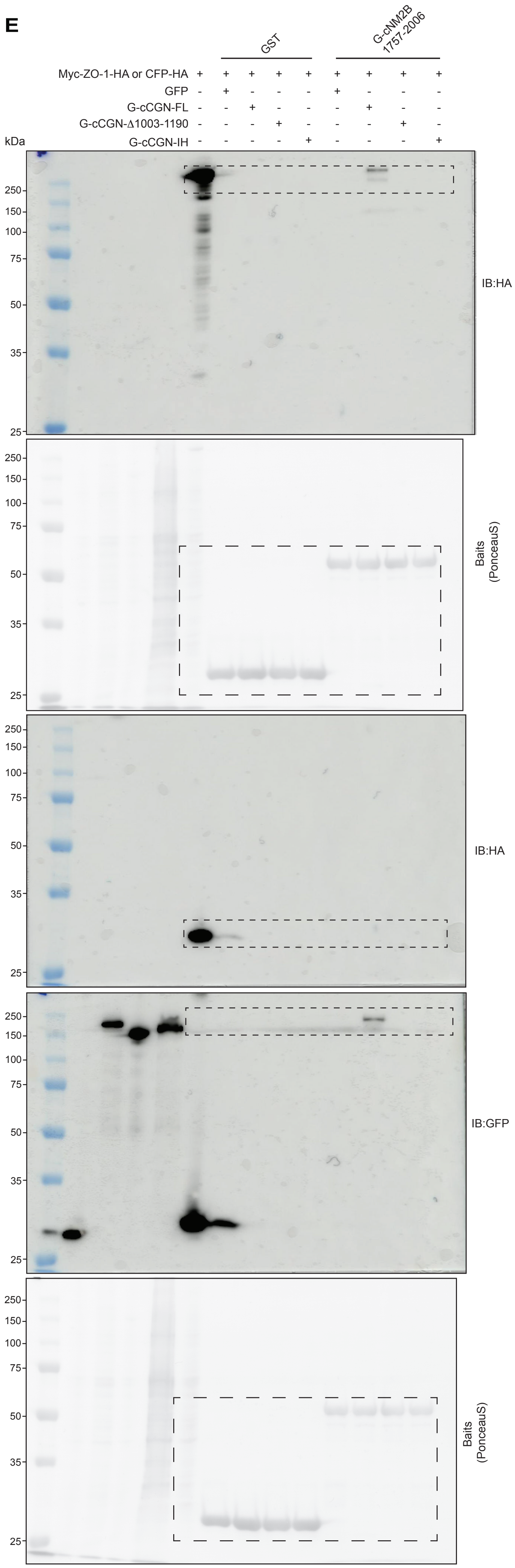

Fig. S7. Blot transparency in Figure S3A-E.

The area used in Figure S3A-E of this study is highlighted with a dashed line box

Journal of Cell Science • Supplementary information

Table S1. Resources Table.

REAGENT or RESOURCE	SOURCE	IDENTIFIER
Antibodies		
Mouse monoclonal anti-GFP (IB, IF)	Roche	Cat# 11814460001, RRID:AB_390913
Rabbit polyclonal anti-HA (IF)	Santa Cruz	Cat# sc-805, RRID:AB_631618
Rabbit polyclonal anti-myc (IF)	Millipore	Cat# 06-549, RRID:AB_310165
Mouse monoclonal GP135 (IF)	BD Biosciences	Cat# MABS1327
Mouse monoclonal anti-cingulin (IB, IF)	Citilab	22BD5A1
Rat polyclonal anti-PLEKHA6 (IF)	(Sluysmans et al., 2021)	RtSZR127
Goat polyclonal anti-ZO-2 (IF)	Santa Cruz	Cat# sc-8148, RRID:AB_2271821
Mouse monoclonal anti-β-tubulin (IB)	Thermo Fisher Scientific	Cat# 32-2600, RRID:AB_2533072
Rabbit polyclonal anti-NMIIB (IB, IF)	Biolegend	Cat# 909901 RRID:AB_291639
Alexa Fluor 488-AffiniPure Donkey Anti-Rabbit IgG	Jackson Laboratory	Cat# 711-545-152, RRID:AB_2313584
Alexa Fluor 488-AffiniPure Donkey Anti-Mouse IgG	Jackson Laboratory	Cat# 715-546-151, RRID:AB_2340850
Cy3-AffiniPure Donkey Anti-Rabbit IgG	Jackson Laboratory	Cat# 711-165-152, RRID:AB_2307443
Cy3-AffiniPure Donkey Anti-Mouse IgG	Jackson Laboratory	Cat# 715-165-151, RRID:AB_2315777
Alexa Fluor 647-AffiniPure Donkey Anti-Goat IgG	Jackson Laboratory	Cat# 705-606-147, RRID:AB_2340438
Cy5-AffiniPure Donkey Anti-Rat IgG	Jackson Laboratory	Cat# 712-175-153, RRID:AB_2340672
Plasmids		
**CGN**		
pCDNA3.1(-)-GFP-cCGN-myc	(Rouaud et al., 2023)	S1115

Journal of Cell Science • Supplementary information

pCDNA3.1(-)-GFP-cCGN-Δ1003-1190-myc	(Rouaud et al., 2023)	S2694
pCDNA3.1(-)-GFP-cCGN-Δ1003-1190+661-1002-myc	This paper	S2831
pCDNA3.1(-)-GFP-cCGN-IH-myc	This paper	S2980
**ZO-1**		
pCDNA3.1(+)- myc-hZO-1-FL-HA	(Spadaro et al., 2017)	S1947
**Control constructs**		
pCDNA3.1(-)- GFP-myc-his	(Guerrera et al., 2016)	S1166
pCDNA3.1(+)- CFP-HA	(Spadaro et al., 2017)	S1150
Recombinant Proteins		
**ZO-1**		
GST-mZO-1-Cter large (1520-1745)	(Vasileva et al., 2022)	S2511
**NM2B**		
GST-cNM2B (1757-2006)	(Rouaud et al., 2023)	S2735
Chemicals, Peptides, Miscellaneous		
Blebbistatin	Sigma-Aldrich	Cat# B0560
Hygromycin B Gold	InvivoGen	Cat# ant-hg-2
Molecular Weight Markers for SDS-PAGE	BioRad	Cat# 1610373
Pierce Protease Inhibitor Tablet, EDTA-free	ThermoScientific	Cat# A32965
Critical Commercial Assays		
jetOPTIMUS	Polyplus	Cat# 117-15
Q5 High fidelity Polymerase	NEB	Cat# M0491L
T4 DNA Ligase	Promega	Cat# M1801
Experimental Models: Cell Lines		
Human embryonic kidney HEK 293T	ATCC	N/A
MDCKII (Madin–Darby Canine Kidney) Tet-off	A Fanning, University of North Carolina	Clontech
MDCKII (Madin–Darby Canine Kidney) Tet-off CGN-KO	(Vasileva et al., 2022)	N/A
MDCKII (Madin–Darby Canine Kidney) Tet-off CGN/CGNL1-double-KO	(Vasileva et al., 2022)	N/A
Experimental Models: Organisms/Strains		
BL21 Competent cells	NEB	Cat# C2530H
DH5 alpha Competent cells	Thermo Fisher	Cat# 18265017
DH10B Competent cells	Thermo Fisher	Cat# 18297010
Software and Algorithms		
Image J	N/A	imagej.nih.gov/ij/ RRID:SCR_003070
Adobe Photoshop	N/A	adobe.com RRID:SCR_014199
Adobe Illustrator	N/A	http://www.adobe.com RRID:SCR_010279
Prism GraphPad	N/A	https://www.graphpad.com/scientific-software/prism/ RRID:SCR_002798
Snapgene Version 3.1.2	N/A	snapgene.com RRID:SCR_015052

Supplementary reference

Guerrera, D., Shah, J., Vasileva, E., Sluysmans, S., Mean, I., Jond, L., Poser, I., Mann, M., Hyman, A. A. and Citi, S. (2016). PLEKHA7 recruits PDZD11 to adherens junctions to stabilize nectins. *J. Biol. Chem.* **291**, 11016-11029. doi:10.1074/jbc.M115.712935

Journal of Cell Science • Supplementary information
